# A comprehensive prediction model for central lymph node metastasis in papillary thyroid carcinoma with Hashimoto’s thyroiditis: BRAF may not be a valuable predictor

**DOI:** 10.3389/fendo.2024.1429382

**Published:** 2024-09-19

**Authors:** Yanwei Chen, Shuangshuang Zhao, Zheng Zhang, Zheming Chen, Bingxin Jiang, Maohui An, Mengyuan Shang, Xincai Wu, Xin Zhang, Baoding Chen

**Affiliations:** Department of Medical Ultrasound, Affiliated Hospital of Jiangsu University, Zhenjiang, Jiangsu, China

**Keywords:** papillary thyroid carcinoma, Hashimoto’s thyroiditis, central lymph node metastasis, prediction model, BRAF mutation, nomogram

## Abstract

**Purpose:**

Papillary thyroid carcinoma (PTC) frequently coexists with Hashimoto’s thyroiditis (HT), which poses challenges in detecting central lymph node metastasis (CLNM) and determining optimal surgical management. Our study aimed to identify the independent predictors for CLNM in PTC patients with HT and develop a comprehensive prediction model for individualized clinical decision-making.

**Patients and methods:**

In this retrospective study, a total of 242 consecutive PTC patients who underwent thyroid surgery and central lymph node dissection between February 2019 and December 2021 were included. 129 patients with HT were enrolled as the case group and 113 patients without HT as control. The results of patients’ general information, laboratory examination, ultrasound features, pathological evaluation, and BRAF mutation were collected. Multivariate logistic regression analysis was used to identify independent predictors, and the prediction model and nomogram were developed for PTC patients with HT. The performance of the model was assessed using the receiver operating characteristic curve, calibration curve, decision curve analysis, and clinical impact curve. In addition, the impact of the factor BRAF mutation was further evaluated.

**Results:**

Multivariate analysis revealed that gender (OR = 8.341, P = 0.013, 95% CI: 1.572, 44.266), maximum diameter (OR = 0.316, P = 0.029, 95% CI: 0.113, 0.888), multifocality (OR = 3.238, P = 0.010, 95% CI: 1.319, 7.948), margin (OR = 2.750, P = 0.046, 95% CI: 1.020, 7.416), and thyrotropin receptor antibody (TR-Ab) (OR = 0.054, P = 0.003, 95% CI: 0.008, 0.374) were identified as independent predictors for CLNM in PTC patients with HT. The area under the curve of the model was 0.82, with accuracy, sensitivity, and specificity of 77.5%, 80.3% and 75.0%, respectively. Meanwhile, the model showed satisfactory performance in the internal validation. Moreover, the results revealed that BRAF mutation cannot further improve the efficacy of the prediction model.

**Conclusion:**

Male, maximum diameter > 10mm, multifocal tumors, irregular margin, and lower TR-Ab level have significant predictive value for CLNM in PTC patients with HT. Meanwhile, BRAF mutation may not have a valuable predictive role for CLNM in these cases. The nomogram constructed offers a convenient and valuable tool for clinicians to determine surgical decision and prognostication for patients.

## 1 Introduction

Thyroid carcinoma (TC) is the predominant malignancy in the endocrine system, originating from thyroid follicular epithelial cells or parafollicular cells, accounting for approximately 3.4% of all newly diagnosed malignancies annually (1). Among these, papillary thyroid carcinoma (PTC), which originates from follicular cells, encompassing over 90% of reported cases (2). The majority of patients exhibit a favorable prognosis due to timely surgical intervention and radioactive iodine therapy, resulting in a 20-year survival rate exceeding 90%. Although the incidence rate of TC has increased yearly, the overall mortality rate of TC patients has remained stable at around 0.2% in the past five decades, hence TC is often characterized as a relatively indolent cancer (3). Nevertheless, approximately 40-90% of the cases experience cervical lymph node metastasis (LNM), mainly including central lymph node metastasis (CLNM) and lateral lymph node metastasis (LLNM), which is identified as a principal risk factor for postoperative recurrence and distant metastasis (4–7). For the PTC patients confirmed by cytopathology, prophylactic central lymph node dissection (pCLND) has been suggested during the surgery. However, as early as 2015, the American Thyroid Association (ATA) has stated that lobectomy alone is sufficient for clinical node-negative (cN0) patients, and a large number of pCLND will inevitably lead to overtreatment (8). Furthermore, the presence of Hashimoto’s thyroiditis (HT) is associated with less advanced PTC and may serve as a protective factor against cancer progression (9). Therefore, accurate preoperative prediction of CLNM in PTC patients with HT is crucial for guiding surgical decisions and improving prognostic accuracy.

Over recent years, the widespread application of high-resolution ultrasonography, facilitated by advancements in ultrasonic technology and equipment, has paralleled an uptrend in PTC incidence (10). Ultrasound assumes a pivotal role in preoperative assessment for PTC patients. However, although ultrasound exhibits high diagnostic value for cervical lymph node metastasis in PTC, its sensitivity in detecting lymph node metastasis in the central neck region is less than 50%, particularly evident in patients with HT (11). Previous studies have observed frequent reactive hyperplasia of cervical lymph nodes in HT patients, with enlarged lymph nodes posing challenges in distinguishing them from LNM on ultrasound imaging (12).

Prior studies have identified certain ultrasound characteristics of PTC as risk factors for CLNM (13). Nevertheless, uneven glandular echoes in patients with HT may affect the ultrasound detection of potential malignant nodules, as these nodules are prone to show irregular or blurry margin (14). The influence of subtle variations in TC ultrasound features on CLNM prediction remains unclear. BRAF mutations are detectable in up to 45% of PTC cases (15). BRAF detection under fine-needle aspiration (FNA) significantly improves the accuracy of papillary thyroid cancer diagnosis, and BRAF mutations correlate closely with larger tumor size, extrathyroidal invasion, multifocality, lymph node metastasis, and advanced staging (16, 17). According to a recent meta-analysis, PTC patients with HT are 55% less likely to have BRAF mutations than PTC patients without HT, but the incidence of multifocal lesions is higher (18). In addition, another study reported that HT is only a protective factor for PTC patients without BRAF but not for patients with BRAF (19). Overall, the role of BRAF mutation in PTC patients with HT remains contentious.

In our study, we retrospectively reviewed consecutive PTC patients with and without HT to identify independent predictors of CLNM. The aim of our study was to develop an accurate and reliable prediction model and to construct a nomogram for evaluating the risk of CLNM for PTC patients with HT, aiming to guide the optimal selection of surgical modality and improve prognostic accuracy for the patients. Additionally, our study further evaluated the impact of BRAF mutation on CLNM in PTC patients with HT.

## 2 Materials and methods

### 2.1 Study design and population

**Figure 1:**
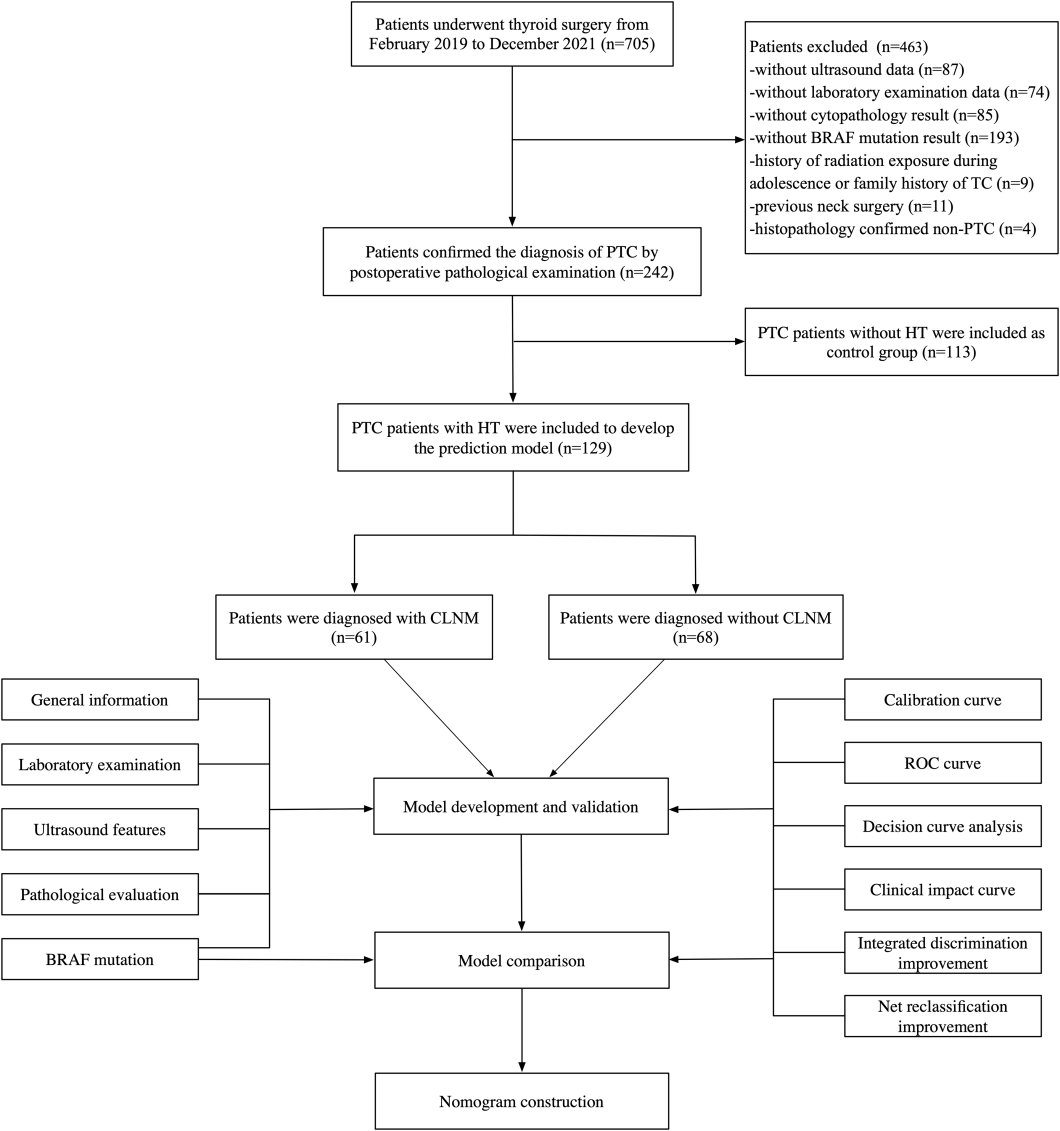
The flowchart of the study, including patient inclusion and exclusion, and statistical analysis. PTC, papillary thyroid carcinoma; HT, Hashimoto’s thyroiditis.

This retrospective study was conducted with the approval of the Ethics Committee of the Affiliated Hospital of Jiangsu University. Patients were admitted to the hospital due to thyroid nodules from February 2019 to December 2021 and informed consents were obtained from all the participants enrolled in the study. All patients underwent high-resolution ultrasound, FNA, and thyroid surgery including subtotal or total thyroidectomy with pCLND. Postoperative pathological analysis confirmed all cases as PTC. Inclusion criteria comprised: (1) ultrasound evidence of malignant features; (2) at least one FNA procedure; (3) absence of prior neck disease. Exclusion criteria included: (1) history of radiation exposure during adolescence or family history of TC; (2) previous neck surgery; (3) without cytopathological results; (4) histopathological findings suggestive of non-PTC; (5) without ultrasound or laboratory examination data. Finally, a total of 242 patients were enrolled in our study (**Figure 1**).

### 2.2 Data collection

Patient data collected included general information, laboratory examination, ultrasound features, pathological evaluation and BRAF mutation. General information contained age and gender of patients. Laboratory examination involved measurement of free triiodothyronine (FT3), free thyroxine (FT4), thyroid-stimulating hormone (TSH), thyroglobulin antibody (TG-Ab), thyroid peroxidase antibody (TPO-Ab), thyroglobulin (TG), thyroid-stimulating hormone receptor antibody (TR-Ab), and calcitonin (CT). Ultrasound features included the location, maximum diameter, markedly hypoechoic, vascularity, aspect ratio, microcalcification, margin and boundary of the nodules. Pathological evaluation included cytopathological and histopathological results. Cytopathological evaluation was interpreted and reported according to the Bethesda System for Reporting Thyroid Cytopathology (TBSRTC), which was further classified into grades 1-6 (20). In our study, grades 1-4 were recorded as negative and 5-6 as positive. The detection of BRAF mutation specifically targeted the BRAFV600E mutation, which is a well-recognized marker for PTC. Postoperative histopathological evaluation was performed by two expert pathologists blinded to other clinical data throughout the study. Multifocality was defined as the presence of two or more PTC lesions within the thyroid gland. Extrathyroidal extension (ETE) was defined as the invasion of surrounding structures, including the strap muscles, trachea, larynx, vasculature, esophagus, and recurrent laryngeal nerve, as observed by intraoperative frozen section evaluation.

### 2.3 Statistical analysis

Statistical analyses were performed using SPSS 26.0 (IBM, New York, USA) and R 4.0.2 (the R Foundation for Statistical Computing, Vienna, Austria). Initially, variables with significant differences (P < 0.05) between the metastatic group (CLNM) and the non-metastatic group (NCLNM) were identified as potential predictors through univariate analysis. Normality of continuous variables was assessed using the Shapiro-Wilk test. Normally distributed continuous variables were presented as mean ± standard deviation and analyzed using independent-samples t-test, while non-normally distributed variables were expressed as median (interquartile range) and analyzed using the Mann-Whitney U test. Categorical variables were presented as frequencies (percentages) and analyzed using the Chi-squared test. Subsequently, a prediction model was established using multivariate logistic regression analysis with a two-way elimination method. When the model has the minimum Akaike information criterion (AIC) value and the model has the best goodness of fit, variables with P < 0.05 are considered as risk factors and included in the model. The performance of the model was evaluated using receiver operating characteristic (ROC) curves and the area under the curve (AUC). Variance inflation factor (VIF) was employed to detect multicollinearity among factors. Internal validation was conducted using the enhanced bootstrap method with 100 resampling iterations. The calibration of the prediction model was assessed using the Hosmer-Lemeshow goodness-of-fit test and calibration plots.

To compare prediction models with and without the factor BRAF mutation, AUC and area under the decision curve (AUDC) were compared using the DeLong test. Decision curve analysis (DCA) and clinical impact curve (CIC) analysis were performed. Additionally, the diagnostic ability of the two models was compared using net reclassification improvement (NRI) and integrated discrimination improvement (IDI). Finally, static and dynamic nomograms were developed to visualize the final prediction model.

## 3 Results

### 3.1 Clinicopathological characteristics of the patients

A total of 242 patients were ultimately included in this retrospective study, including 129 patients with HT and 113 patients without HT. The clinical baseline information of patients is summarized in **Table 1**. Among the patients with HT, 61 patients (47.3%) developed CLNM (CLNM group), while 68 patients (52.7%) did not (NCLNM group). The cohort comprised 16 male patients (12.4%) and 113 female patients (87.6%). In the NCLNM group, there were 3 male patients (4.4%) and 65 female patients (95.6%), while in the CLNM group, there were 13 male patients (21.3%) and 48 female patients (78.7%). Gender distribution between the two groups showed a significant difference (P = 0.004). The median age of patients in the NCLNM group was 45 years (interquartile range, IQR: 18.75), and in the CLNM group, it was 44 years (IQR: 22.50), with no statistical difference observed between the groups (P = 0.755).

**Table 1:** Comparison of the clinicopathological characteristics between CLNM and NCLNM group of the PTC patients with and without HT.

Variable	Group	HT				Non-HT			
		Cases (n=129)	CLNM (n=61)	NCLNM (n=68)	P	Cases (n=113)	CLNM (n=67)	NCLNM (n=46)	P
General information									
Age (years)			44.00 (22.50)	45.00 (18.75)	0.755		40.94 (11.89)	47.15 (13.19)	**0.010**
	<55	95	45 (73.8)	50 (73.5)	0.975	88	49 (73.1)	39 (84.8)	0.143
	≥55	34	16 (26.2)	18 (26.5)		25	18 (26.9)	7 (15.2)	
Gender					**0.004**				0.726
	Male	16	13 (21.3)	3 (4.4)		34	21 (31.3)	13 (28.3)	
	Female	113	48 (78.7)	65 (95.6)		79	46 (68.7)	33 (71.7)	
Laboratory examination									
FT3 (pmol/L)			4.71 (0.77)	4.71 (0.97)	0.567		4.96 ± 0.75^¶^	4.90 ± 0.64^¶^	0.626
FT4 (pmol/L)			15.30 ± 4.59^¶^	14.44 ± 3.77^¶^	0.245		16.90 ± 2.44^¶^	16.02 ± 2.32^¶^	0.055
TSH (μIU/mL)			2.09 (1.74)	2.09 (2.09)	0.914		1.74 (1.01)	1.74 (0.97)	0.919
TG-Ab (IU/mL)			225.80 (329.49)	244.10 (422.20)	0.932		20.36 (19.17)	18.69 (11.74)	0.640
TPO-Ab (IU/mL)			95.11 (301.61)	216.20 (409.74)	0.204		4.96 (7.73)	2.46 (8.85)	0.083
TG (ng/mL)			4.30 (11.70)	7.90 (18.28)	0.256		36.20 (67.14)	32.64 (52.59)	0.312
TR-Ab (U/L)			0.63 (0.59)	0.85 (0.57)	**0.009**		0.63 (0.67)	0.58 (0.35)	0.660
CT (pg/ml)			5.35 (1.69)	5.35 (1.57)	0.504		6.36 (3.86)	6.94 (5.15)	0.813
Ultrasound features									
TI-RADS					0.186				0.216
	3	5	4 (6.6)	1 (1.5)		1	1 (1.5)	0 (0)	
	4a	64	26 (42.6)	38 (55.9)		55	28 (41.8)	27 (58.7)	
	4b	41	19 (31.1)	22 (32.4)		45	28 (41.8)	17 (37.0)	
	4c	8	4 (6.6)	4 (5.9)		9	8 (11.9)	1 (2.2)	
	5	11	8 (13.1)	3 (4.4)		3	2 (3.0)	1 (2.2)	
Maximum diameter (mm)					**< 0.001**				**0.013**
	≤10	68	22 (36.1)	46 (67.6)		76	39 (58.2)	37 (80.4)	
	>10	61	39 (63.9)	22 (32.4)		37	28 (41.8)	9 (19.6)	
Aspect ratio					**0.013**				0.405
	<1	80	31 (50.8)	49 (72.1)		52	33 (49.3)	19 (41.3)	
	≥1	49	30 (49.2)	19 (27.9)		61	34 (50.7)	27 (58.7)	
Microcalcification					0.426				**0.012**
	No	36	15 (24.6)	21 (30.9)		60	29 (43.3)	31 (67.4)	
	Yes	93	46 (75.4)	47 (69.1)		53	38 (56.7)	15 (32.6)	
Boundary					0.765				0.815
	Clear	68	33 (54.1)	35 (51.5)		58	35 (52.2)	23 (50.0)	
	Unclear	61	28 (45.9)	33 (48.5)		55	32 (47.8)	23 (50.0)	
Margin					**0.027**				0.720
	Smooth	42	14 (23.0)	28 (41.2)		69	40 (59.7)	29 (63.0)	
	Irregular	87	47 (77.0)	40 (58.8)		44	27 (40.3)	17 (37.0)	
Markedly hypoechoic					0.166				0.167
	No	101	51 (83.6)	50 (73.5)		58	38 (56.7)	20 (43.5)	
	Yes	28	10 (16.4)	18 (26.5)		55	29 (43.3)	26 (56.5)	
Vascularity					0.114				0.146
	Sparse	97	42 (68.9)	55 (80.9)		46	31 (46.3)	15 (32.6)	
	Abundant	32	19 (31.1)	13 (19.1)		67	36 (53.7)	31 (67.4)	
Location					0.129				0.587
	Upper	38	23 (37.7)	15 (22.1)		34	17 (25.4)	17 (37.0)	
	Middle	41	14 (23.0)	27 (39.7)		56	36 (53.7)	20 (43.5)	
	Lower	45	22 (36.1)	23 (33.8)		17	10 (14.9)	7 (15.2)	
	Isthmus	5	2 (3.3)	3 (4.4)		6	4 (6.0)	2 (4.3)	
Pathology and BRAF									
TBSRTC					**0.032**				0.345
	Negative	37	12 (19.7)	25 (36.8)		9	4 (6.0)	5 (10.9)	
	Positive	92	49 (80.3)	43 (63.2)		104	63 (94.0)	41 (89.1)	
BRAF mutation					0.148				0.118
	Negative	26	9 (14.8)	17 (20.6)		39	27 (40.3)	12 (26.1)	
	Positive	103	52 (85.2)	51 (79.4)		74	40 (59.7)	34 (73.9)	
Extrathyroidal extension					**0.039**				**0.007**
	No	103	44 (72.1)	59 (86.8)		64	31 (46.3)	33 (71.7)	
	Yes	26	17 (27.9)	9 (13.2)		49	36 (53.7)	13 (28.3)	
Multifocality					**0.001**				**0.002**
	No	77	27 (44.3)	50 (73.5)		56	25 (37.3)	31 (67.4)	
	Yes	52	34 (55.7)	18 (26.5)		57	42 (62.7)	15 (32.6)	

CLNM, central lymph node metastasis; NCLNM, no central lymph node metastasis; PTC, papillary thyroid carcinoma; HT, Hashimoto’s thyroiditis; TSH, thyrotropin; FT3, free triiodothyronine; FT4, free thyroxine; TG-Ab, thyroglobulin antibody; TPO-Ab, thyroid peroxidase antibody; TG, thyroglobulin; TR-Ab, thyroid-stimulating hormone receptor antibody; CT, calcitonin; TBSRTC, The Bethesda System for Reporting Thyroid Cytopathology.

Except where indicated, data are presented as frequency (percentage) or median (interquartile spacing).

^¶^The data are presented as means ± SD (standard deviation).

Bold P values indicate statistical significance (P < 0.05).

Regarding laboratory indicators, a significant difference was found between the two groups in TR-Ab, with values of 0.85 U/L (IQR: 0.57) in the NCLNM group and 0.63 U/L (IQR: 0.59) in the CLNM group (P = 0.009). However, no significant differences were observed in levels of FT3 (P = 0.567), FT4 (P = 0.245), TSH (P = 0.914), TG-Ab (P = 0.932), TG (P = 0.256), TPO-Ab (P = 0.204), and CT (P = 0.504) between the two groups.

Among ultrasound features of thyroid nodules, statistically significant differences were observed in maximum diameter (P < 0.001), aspect ratio (P = 0.013), and margin (P = 0.027) between the two groups. However, no significant differences were found in calcification (P = 0.426), boundary (P = 0.765), markedly hypoechoic (P = 0.166), vascularity (P = 0.114), and location (P = 0.129).

Regarding pathological evaluation and BRAF mutation, TBSRTC (P = 0.032), multifocality (P = 0.001), and extrathyroidal extension (ETE) (P = 0.039) showed significant differences between the two groups, while BRAF mutation did not (P = 0.148).

Additionally, 113 patients without HT were included in the study as control. Among them, 67 patients (59.3%) developed CLNM (CLNM group), while 46 patients (40.7%) did not (NCLNM group). The results showed that age (P = 0.010), maximum diameter (P = 0.013), microcalcification (P = 0.012), ETE (P = 0.007) and multifocality (P = 0.002) were significant different between the CLNM and NCLNM groups.

### 3.2 Development of the prediction model

The aforementioned results identified eight candidate variables significantly associated with CLNM among patients with HT: gender, TR-Ab, maximum diameter, aspect ratio, multifocality, margin, cytopathology, and ETE. These variables were included in multivariate logistic regression analysis to develop the prediction model. The continuous variable TR-Ab, which did not follow a normal distribution, was logarithmically transformed (log10) to achieve normality before analysis. Multivariate analysis revealed that gender, maximum diameter, multifocality, margin, and TR-Ab were significant independent predictors for CLNM in the final model (**Table 2**).

**Table 2:** Multivariate analysis of factors associated with CLNM in PTC patients with HT.

Variable	Group	Cases	OR	95% CI		P
				Lower	Upper	
Gender						
	Male	16	8.341	1.572	44.266	**0.013**
	Female	113	1.000			
Maximum diameter						
	≤10	68	0.316	0.113	0.888	**0.029**
	>10	61	1.000			
Aspect ratio						
	<1	80	1.000			
	≥1	49	2.020	0.728	5.610	0.177
Multifocality						
	No	77	1.000			
	Yes	52	3.238	1.319	7.948	**0.010**
Margin						
	Smooth	42	1.000			
	Irregular	87	2.750	1.020	7.416	**0.046**
TBSRTC						
	Negative	37	1.000			
	Positive	92	2.607	0.883	7.703	0.083
Extrathyroidal extension						
	No	103	1.000			
	Yes	26	2.416	0.783	7.455	0.125
TR-Ab						
		129	0.054	0.008	0.374	**0.003**

CLNM, central lymph node metastasis; PTC, papillary thyroid carcinoma; HT, Hashimoto’s thyroiditis; TBSRTC, The Bethesda System for Reporting Thyroid Cytopathology; TR-Ab, thyroid-stimulating hormone receptor antibody.

The data of TR-Ab were logarithm transformed to meet the normal distribution. Bold P values indicate statistical significance (P < 0.05).

### 3.3 Evaluation and validation of the prediction model

**Figure 2:**
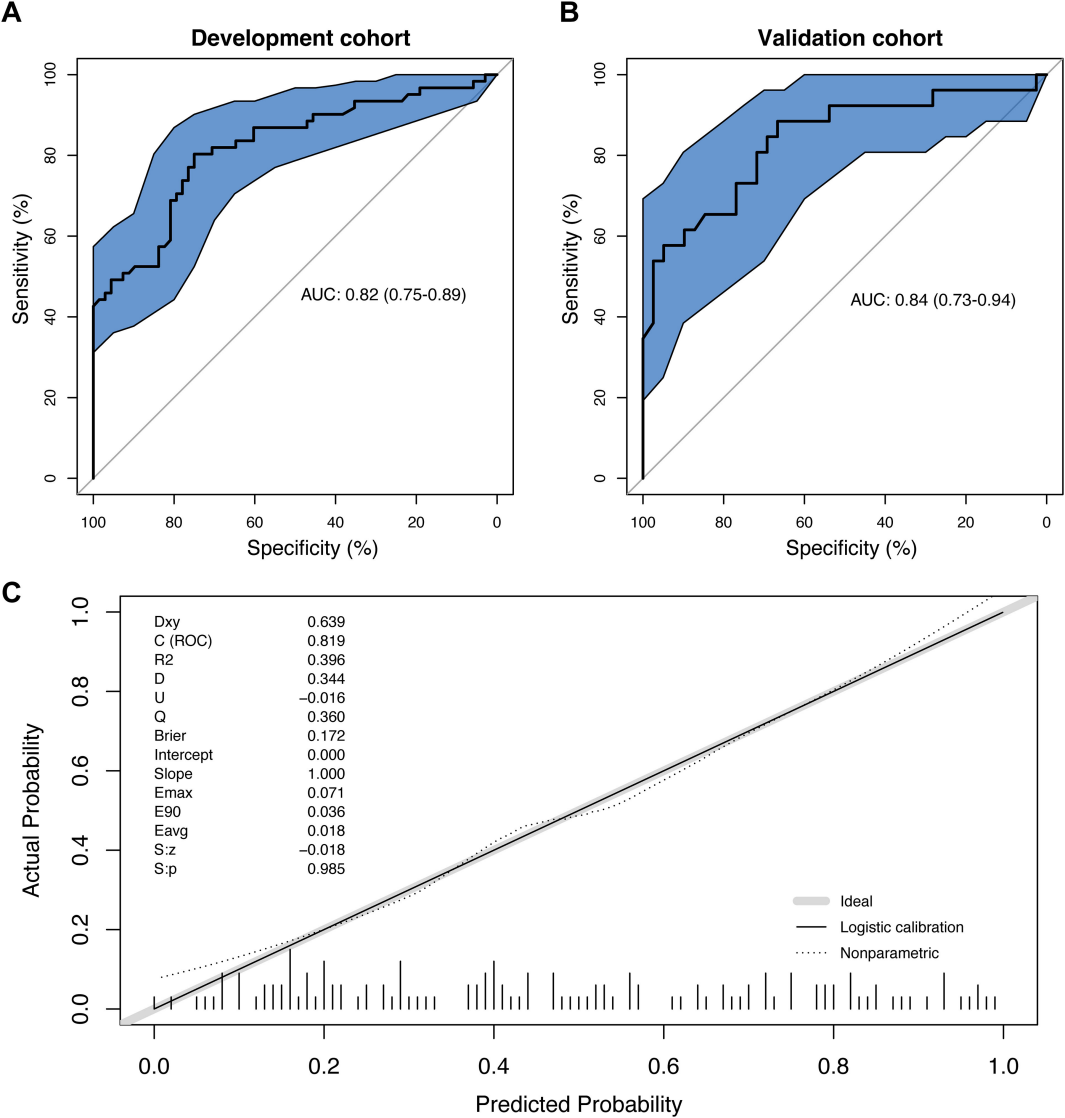
The evaluation of the prediction model. **(A)** The receiver operating characteristics (ROC) curve and area under the curve (AUC) in the development cohort; **(B)** The ROC curve and AUC in the validation cohort; **(C)** The calibration curve derived from the 100 resampling bootstrap analysis.

The ROC curve of the prediction model was plotted (**Figure 2A**). The AUC value of the prediction model was 0.82 (95% CI: 0.75, 0.89), with an accuracy rate of 77.5%. The sensitivity and specificity of the model were 80.3% and 75.0%, respectively. Moreover, the positive predictive value and negative predictive value were 74.2% and 81.0%, respectively.

Then, 65 patients (50% of the development cohort) were randomly selected as the internal validation cohort. The AUC value of the validation cohort was 0.84 (95% CI: 0.73, 0.894) (**Figure 2B**). The repeatability of the model development process was tested through the internal validation of the model, and the enhanced bootstrap method was used to draw the calibration curve of the model with 100 resampling iterations. According to Hosmer-Lemeshow goodness-of-fit test, the chi-square statistic was 12.40 (P=0.19), suggesting that the calibration of the model was perfect (**Figure 2C**).

### 3.4 Exploring the impact of including BRAF mutation into the prediction model

Although there was no statistical significance in BRAF mutation between the CLNM and NCLNM groups (P=0.148) in the previous results, BRAF mutation remains a pivotal factor in the progression of PTC, as evidenced by previous studies and clinical practice. Therefore, our study attempted to investigate whether the inclusion of BRAF mutations as a predictor can improve the predictive efficacy of the model has been constructed.

**Figure 3:**
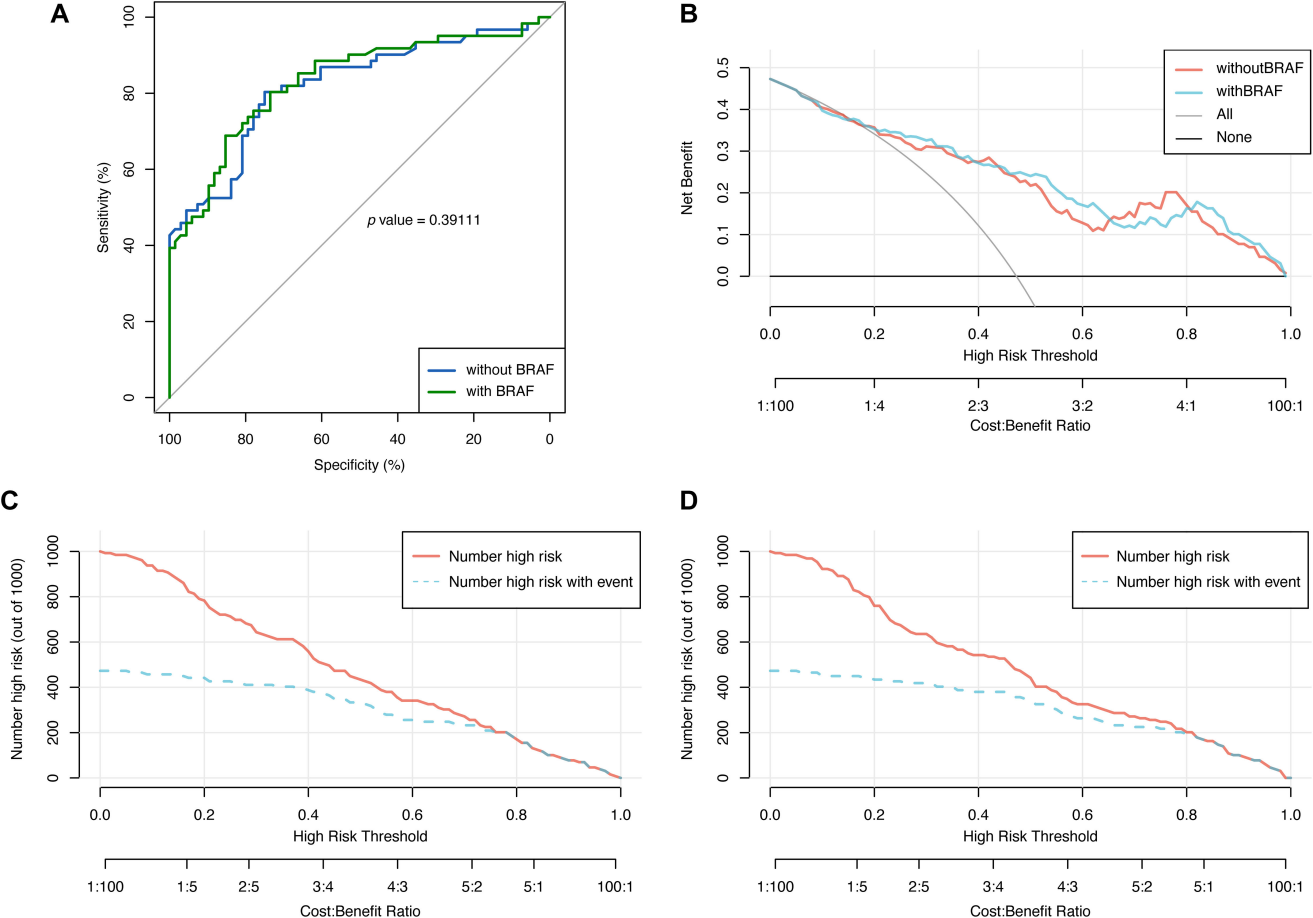
The comparison of the models with and without BRAF mutation via receiver operating characteristics (ROC) curve, decision curve and clinical impact curve. **(A)** ROC curve was used to evaluate the performance of the models with and without BRAF mutation. **(B)** Decision curve was used to evaluate the net benefit of the models with and without BRAF mutation. Clinical impact curve was used for assessing the clinical effective rates of the model without **(C)** and with BRAF mutation **(D)**.

Statistical results shown that the AUC of the model with BRAF mutation (referred to as the new model) was 0.83 (95% CI: 0.76, 0.90), slightly higher than that of the model without BRAF mutation (referred to as the old model), which was 0.82 (95% CI: 0.75, 0.89). However, the Delong test yielded Z=-0.85761 and P=0.39, indicating no statistically significant difference between the AUCs of the two models (**Figure 3A**). Additionally, decision curves for both models were plotted, revealing an area under the decision curve of 0.23 for the old model and 0.24 for the new model. Analysis of the decision curves did not demonstrate that the new model yielded greater net clinical benefits compared to the old model (**Figure 3B**). Similarly, the clinical impact curves of the two models did not illustrate greater clinical effective rates in the new model (**Figures 3C, D**).

To further evaluate the reclassification performance of the new model compared with the old model, the reclassification indicators that can quantify this degree, i.e. net reclassification improvement (NRI) and integrated discrimination improvement (IDI), were calculated. The categorical NRI, continuous NRI, and IDI were 0.0017 (95% CI: -0.086, 0.089, P=0.97), 0.2637 (95% CI: -0.014, 0.542, P=0.06), and 0.0259 (95% CI: -0.001, 0.053, P=0.06), respectively, which further indicated that the performance was not significantly improved after the integration of the BRAF into the model.

### 3.5 Construction of the nomogram for central lymph node metastasis

**Figure 4:**
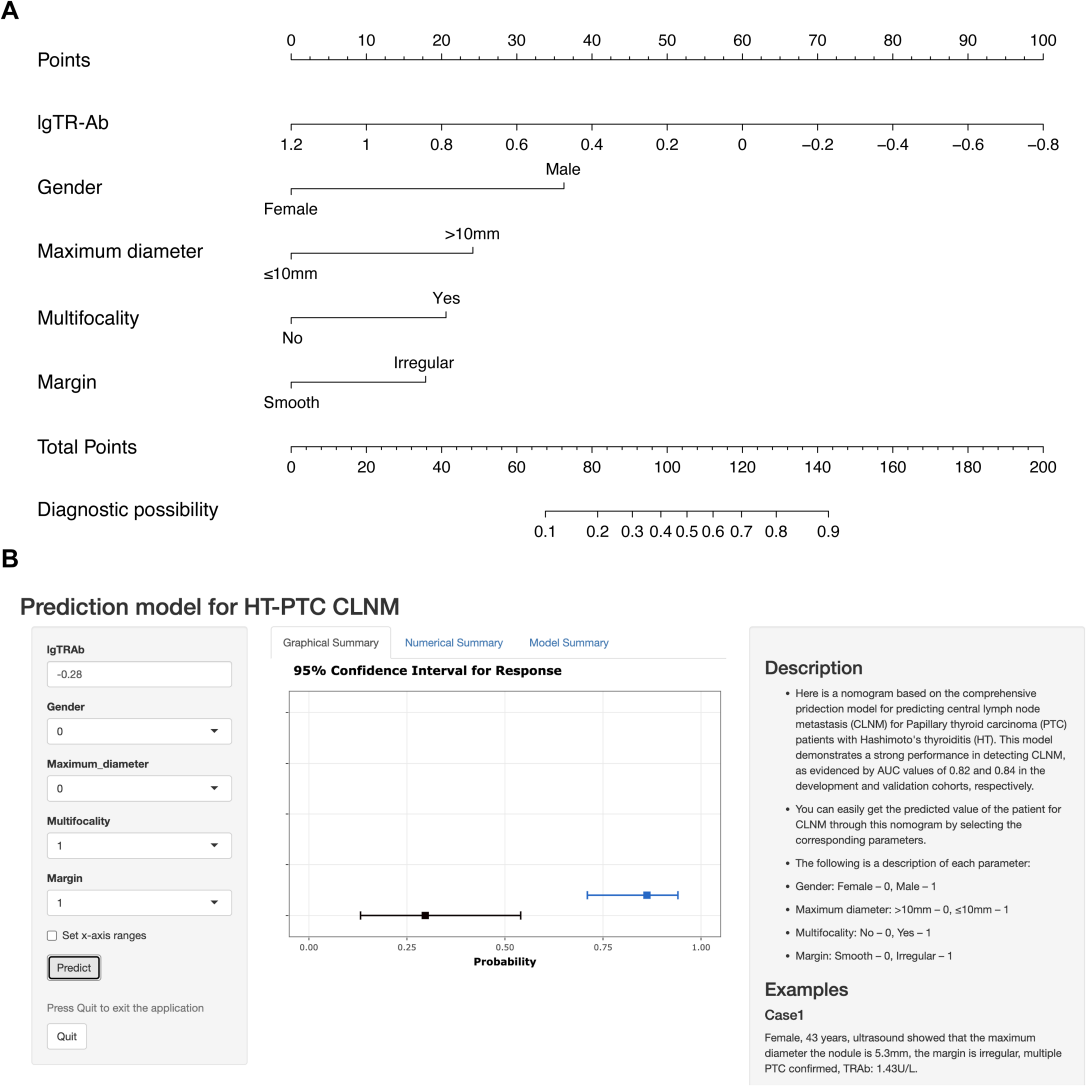
The nomogram constructed for predicting CLNM in PTC patients with HT. **(A)** the static nomogram; **(B)** the dynamic nomogram online (https://cywujs.shinyapps.io/htptc/).

Multivariable analysis demonstrated that gender, maximum diameter, multifocality, margin and TR-Ab were independent predictors of CLNM in PTC patients with HT. A nomogram based on these predictors was developed to visually represent the prediction model (**Figure 4A**). Moreover, leveraging the static nomogram, we established a dynamic nomogram online (https://cywujs.shinyapps.io/htptc/) for convenient generation of predicted values of CLNM for PTC patients with HT (**Figure 4B**).

## 4 Discussion

Thyroid carcinoma ranks as the most common malignant tumor of the endocrine system, with papillary thyroid carcinoma (PTC) comprising the majority (21, 22). While PTC generally exhibits a favorable prognosis as a well-differentiated papillary carcinoma, lymph node metastasis remains prevalent among patients, serving as the primary risk factor for recurrence (23). Notably, Hashimoto’s thyroiditis (HT), characterized by lymphocytic infiltration of the thyroid gland causing central cervical lymph node enlargement, poses a challenge in distinguishing it from central lymph nodes metastasis (CLNM) preoperatively (24). Several studies have indicated a negative correlation between HT and lymph node metastasis in PTC. Patients with HT demonstrate more favorable pathological characteristics and prognoses, including higher rate of clinical remission and longer recurrence-free survival (25–30).

However, the ultrasound features of CLNM present a diagnostic challenge due to the complex anatomy close to the thoracic. According to previous research, the general rate of occult CLNM in PTC has been reported up to 82% (31–33). However, the results of previous studies have shown that the impact of lymph node metastasis (LNM) on the long-term prognosis of PTC patients remains controversial. Based on the analysis of the Surveillance, Epidemiology, and End Results (SEER) database by Liu et al., in some young and middle-aged PTC patients, LNM may disappear spontaneously, and the tumor may become smaller (34). In contrast, other studies shown that progressive lymph node burden independently increases the risk of distant metastasis of PTC (35, 36). Moreover, CLNM may associated with compromised survival in young patients (37). Consequently, several institutions opt for prophylactic central cervical lymph node dissection (pCLND) in PTC patients, even in the absence of imaging evidence. However, this approach carries the risk of complications such as recurrent laryngeal nerve injury, hypoparathyroidism, and chyle leakage (38–41). Therefore, accurate preoperative prediction model for CLNM at the time of initial surgery is imperative to guide clinicians in selecting the optimal therapeutic strategy and ultimately improve the outcomes of patients.

In this retrospective study, we enrolled 129 PTC patients with HT and conducted a comprehensive statistical analysis to establish a prediction model for CLNM. Univariate analysis revealed that gender, TR-Ab, maximum diameter, aspect ratio, margin, cytopathology, multifocality, ETE and multifocality were significantly correlated with CLNM among PTC patients with HT, which was different from the finding of the control group that consisted of 113 PTC patients without HT. On multivariate analysis, gender, maximum diameter, multifocality, margin and TR-Ab were independent predictors for CLNM. Subsequently, a prediction model for CLNM was constructed based on these predictors, achieving an AUC of 0.82 in the ROC curve analysis. Moreover, we developed a nomogram to visually represent the model, facilitating clinicians in predicting the risk of CLNM for PTC patients with HT.

Among the general information of patients, gender emerged as an independent predictor (P=0.01), consistent with findings from a previous meta-analysis by Mao et al. (42). Despite the higher incidence of PTC in females compared to males, our results suggest that male PTC patients may present with more aggressive features (43). Notably, age did not show significant differences between the CLNM and NCLNM groups in our study. Previous research by Du et al. identified age over 45 years as a significant independent predictor of CLNM, whereas Awny et al. reported a higher likelihood of CLNM in patients younger than twenty years (44, 45). Thus, the impact of age on CLNM remains controversial and warrants further investigation.

While ultrasound is a commonly utilized method for detecting CLNM, its effectiveness is limited due to the presence of certain lymph nodes in deep anatomical locations near the trachea and surrounding structures (6, 46). In fact, PTC patients with HT have a larger number of cervical lymph node enlargements, which can be seen in about 23% of patients and bring certain challenges to the preoperative ultrasound scanning for CLNM (47). Therefore, more and more studies have tried to investigate the ultrasound characteristics of PTC to predict CLNM. In a previous study by Chen et al., which included 133 PTC patients with HT, significant statistical differences were found in nodule size (P<0.001), aspect ratio (P=0.019), and calcification (P=0.046) between patients with and without CLNM. Further multivariate analysis revealed that nodules larger than 10 mm were considered a risk factor for CLNM (P<0.001) (48). Our study corroborates these findings, with maximum diameter (P<0.01) and margin (P=0.03) emerging as independent predictors for CLNM. Interestingly, certain factors deemed important in previous studies were not included in our prediction model. For instance, some studies have suggested that tumors located in the upper pole of the thyroid are more prone to CLNM due to the rich blood supply and lymphatic drainage in this region, or due to continuous physical pressure from adjacent thyroid cartilage (49, 50). However, our study found no significant difference in tumor location between the CLNM and NCLNM groups (P=0.129), consistent with the results reported by Yu et al. (P=0.357) (51).

Thyroid peroxidase antibody (TPO-Ab) and thyroglobulin antibody (TG-Ab) are autoantibodies targeting thyroid antigens and are considered important clinical markers for Hashimoto’s thyroiditis (HT), with positivity rates of approximately 75% and 90% in HT patients, respectively (52). Noel et al. evaluated the significance of TPO-Ab and TG-Ab levels associated with lymph node metastases (LNM) in patients with differentiated thyroid carcinoma (DTC), finding that preoperative TG-Ab was an independent predictor of LNM (53). However, a meta-analysis by Zhang et al. suggested that the presence of TPO-Ab is associated with an increased prevalence of DTC, while its effectiveness as a prognostic marker for DTC patients requires further investigation (54). In our study, TPO-Ab (P=0.204) and TG-Ab (P=0.932) did not show statistically significant differences in CLNM between the CLNM and NCLNM groups. Notably, thyroid stimulating hormone (TSH) has been confirmed to be a growth factor that affects the occurrence or progression of PTC (55, 56). In contrast to previous study by Liu et.al, our results revealed that TSH was not an independent predictor for PTC patients with HT (57). In view of this, we tried to include TSH into the prediction model as a predictor variable to improve its performance. However, the new model did not show better predictive ability than the old model (**Supplementary Material 1**). Unexpectedly, thyroid-stimulating hormone receptor antibody (TR-Ab), a hallmark for thyrotoxicosis and assisting in the diagnosis of Graves’ disease, emerged as an independent predictor according to our results (58). TR-Ab can be further divided into two types, including stimulating (TS-Ab) and blocking (TB-Ab) which can be transformed into each other, the increased level of TR-Ab can be also detected in HT patients (59, 60). Previous studies have reported that the thyroid-stimulating hormone receptor (TSHR) is expressed not only in thyrocytes but also in TC cells, exerting significant effects on TC occurrence, development, and immune evasion (61, 62). Our results indicated that TR-Ab (P=0.009) was negatively correlated with CLNM. Of note, while significant differences were observed, most patients’ TR-Ab levels remained within the normal range (0-1.5U/L), and only 5 cases in the NCLNM group (5/68, 7.35%) and 1 case in the CLNM group (1/61, 1.64%) exhibited the elevation of TR-Ab levels. We speculate that the inhibitory subtype of TR-Ab may block the binding of TSHR to TSH on PTC cells, thereby reducing the activation effect of TSH on them. The specific underlying mechanism remains to be further explored.

The BRAF mutation is widely recognized as a pivotal factor in the development and progression of PTC. Previous studies have suggested that patients harboring the BRAF mutation are closely associated with aggressive pathological features, including LNM, extrathyroidal extension, and advanced disease stages (63–65). Additionally, it correlates with the overexpression of tumor-promoting factors such as VEGF and MET, and with more aggressive tumor variants like the tall cell variant of PTC. However, its utility as a prognostic marker is debated due to its low specificity in predicting disease recurrence (66). Meanwhile, it was also revealed that for newly diagnosed well-differentiated thyroid carcinoma, BRAF does not independently predict the risk of cancer-related mortality (67). In our study, the results did not reveal a significant difference in BRAF mutation status between the groups (P=0.148). Despite the essential role of BRAF, when artificially included as a predictor in our prediction model, the new model did not demonstrate significant differences from the original model in terms of various evaluation metrics, including AUC, AUDC, CIC, NRI and IDI. These findings further suggest that BRAF mutation status may not be related to CLNM in PTC patients with HT in our study. A recent study by Zhao et al. on the correlation between BRAF mutation and lymph node metastasis and recurrence of papillary thyroid microcarcinoma also reported similar results (68). This finding necessitates a reconsideration of current clinical practices that rely on BRAF status for risk assessment and treatment decisions. Comprehensive evaluation should be carried out by focusing on a broader range of predictors to develop personalized treatment plans, rather than relying excessively on BRAF. In addition, studies with larger sample sizes are needed to verify the limited role of BRAF in predicting CLNM in PTC patients with HT, promoting more evidence-based management strategies. This transformation will improve the accuracy of risk stratification, improve patient prognosis, and optimize resource utilization.

Among the pathological predictors, multifocality (P=0.001) and extrathyroidal extension (ETE) (P=0.039) exhibited significant differences between the two groups. Tumor multifocality is common in PTC patients, with a prevalence ranging from 18% to 87% (69). Our results indicated that multifocality (P<0.01) was an independent predictor for CLNM in PTC patients with HT, in agreement with earlier studies (70, 71). ETE has long been regarded as an unfavorable factor for DTC and previous research conducted by Bortz et al. showed that all levels of ETE were significantly associated with lymph node and distant metastasis (72). Although univariate analysis identified ETE as being associated with CLNM, there was no statistical significance in multivariate analysis in our study.

Despite the promising findings of our study, several limitations should be acknowledged. Firstly, due to the problems of image quality and the replacement of laboratory index standards, the cases in early years cannot be well included in the statistics, limiting the total volume of samples. Secondly, while the prediction model was internally validated in our study, external validation using data from other centers was lacking, which limits the assessment of its universality and applicability in different populations. Future research should focus on validating the model using independent datasets from different institutions to confirm its generalizability and robustness. Lastly, the ultrasound technology utilized in this study, including two-dimensional ultrasound and color Doppler flow imaging, did not incorporate newer ultrasound techniques such as contrast-enhanced ultrasound and elastography, which may have different characteristics relevant to CLNM.

In summary, our findings indicate that male, maximum diameter > 10mm, multifocal tumors, irregular margin and lower TR-Ab level are significantly associated with CLNM in PTC patients with HT. The developed nomogram provides a valuable tool for accurately predicting the risk of CLNM in these patients, aiding clinicians in decision-making regarding pCLND and providing important clinical reference for the prognosis of patients.

## Data Availability

The original contributions presented in the study are included in the article/**Supplementary Material**. Further inquiries can be directed to the corresponding author.
