# Supplementary material for: A comprehensive prediction model for central lymph node metastasis in papillary thyroid carcinoma with Hashimoto’s thyroiditis: BRAF may not be a valuable predictor
Source: Front Endocrinol (Lausanne). 2024 Sep 19;15:1429382. doi: 10.3389/fendo.2024.1429382 (PMC11446765; doi:10.3389/fendo.2024.1429382)
Supplement: Supplementary file 2 [file DataSheet2.docx]

Supplementary Material

# Exploring the impact of including TSH into the prediction model

In our study, TSH did not show statistically significance between the CLNM and NCLNM groups (P=0.914). Despite this, TSH is recognized as a growth factor influencing the occurrence and progression of PTC, prompting us to include it into the model to potentially enhance its predictive ability.

The model incorporating TSH (referred to as the new model) had an AUC of 0.83 (95% CI: 0.66, 0.87), slightly higher than the AUC of 0.82 (95% CI: 0.75, 0.89) for the model without TSH (referred to as the old model). However, the Delong test yielded Z=-0.80181 and P=0.42, indicating no statistically significant difference between the AUCs of the two models **(Supplementary Figure 1A)**. Furthermore, decision curves showed an area under the decision curve of 0.23 for the old model and 0.24 for the new model, with no significant difference in net clinical benefits between them **(Supplementary Figure 1B)**. Similarly, the clinical impact curves also did not demonstrate a higher clinical effectiveness rate for the new model **(Supplementary Figures 1C and 1D)**.

To further assess the new model's reclassification performance compared to the old model, the net reclassification improvement (NRI) and integrated discrimination improvement (IDI) were calculated. The categorical NRI was -0.0311 (95% CI: -0.106, 0.044, P=0.41), and the IDI was 0.0135 (95% CI: -0.007, 0.034, P=0.20), indicating that the integration of TSH into the model did not significantly enhance its performance.

# Supplementary Figure 1


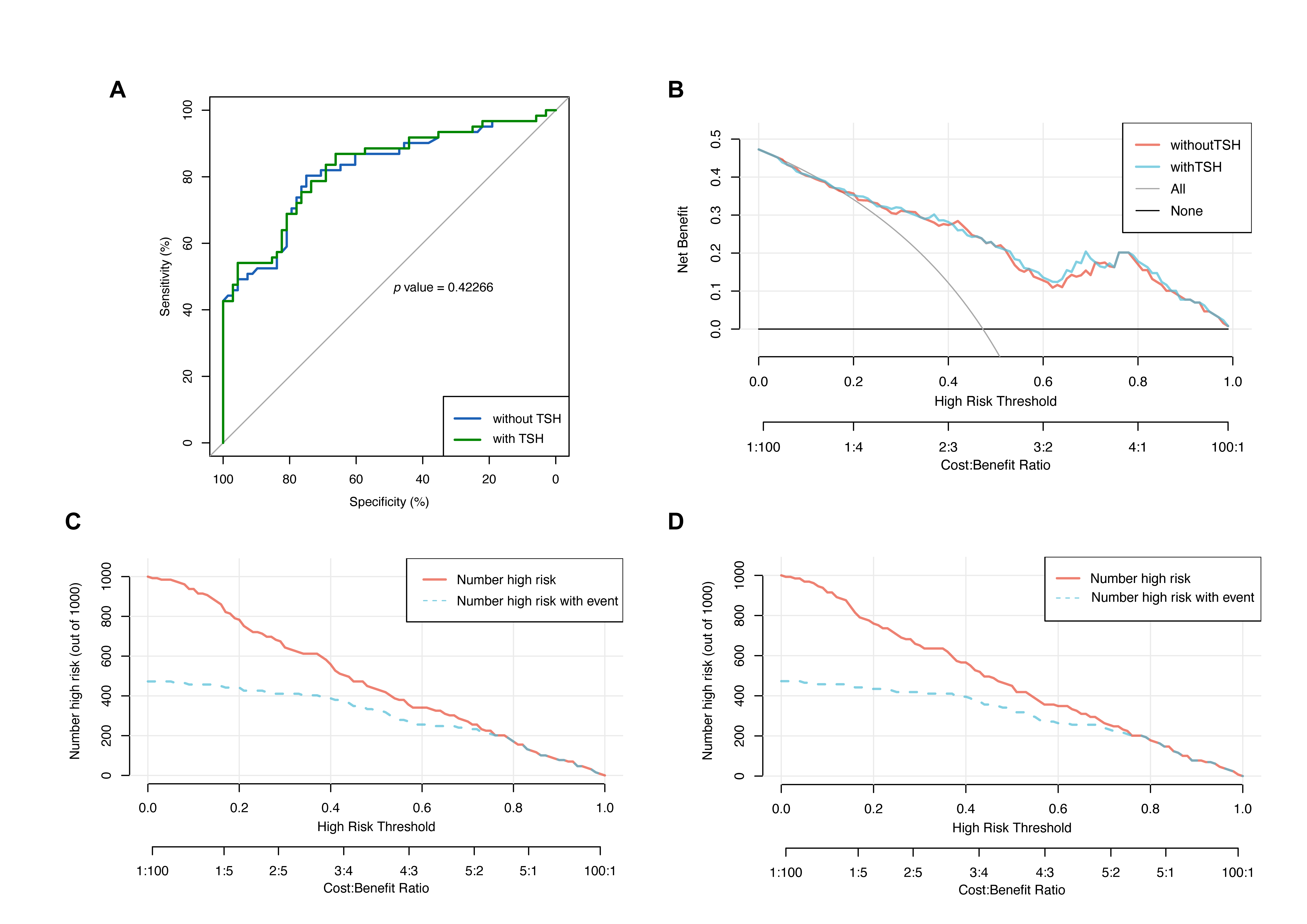


**Supplementary Figure 1.** The comparison of the models with and without TSH via receiver operating characteristics (ROC) curve, decision curve and clinical impact curve. **(A)** ROC curve was used to evaluate the performance of the models with and without TSH. **(B)** Decision curve was used to evaluate the net benefit of the models with and without TSH. Clinical impact curve was used for assessing the clinical effective rates of the model without **(C)** and with TSH **(D)**.
